# Changes of methylation at enhancers appear to be essential for HIV infection progression

**DOI:** 10.1186/s13148-025-01910-3

**Published:** 2025-06-19

**Authors:** Olga Taryma-Leśniak, Jan Bińkowski, Kaja Mielczak, Bogusz Aksak-Wąs, Malwina Karasińska-Cieślak, Marta Sobalska-Kwapis, Dominik Strapagiel, Miłosz Parczewski, Tomasz Kazimierz Wojdacz

**Affiliations:** 1 Independent Clinical Epigenetics Laboratory, Pomeranian Medical University in Szczecin, Szczecin, Poland; 2 Regional Centre for Digital Medicine, Pomeranian Medical University in Szczecin, Szczecin, Poland; 3 Department of Infectious, Tropical Diseases and Immune Deficiency, Pomeranian Medical University in Szczecin, Szczecin, Poland; 4 Centre for Digital Biology and Biomedical Science – Biobank Lodz, Faculty of Biology and Environmental Protection, University of Lodz, Lodz, Poland

**Keywords:** Methylation, HIV, Progression, Epigenetics

## Abstract

**Background:**

We studied the influence of the European HIV-1 subtype B (most common in the Western and Central Europe) and subtype A6 (prevalent in Eastern Europe including Ukraine and Russia) on host methylome during infection progression and in virus-subtype-specific manner.

**Results:**

Our results show that regardless of virus subtype, in the initial phase of the infection, HIV-related methylation changes more frequently affect parts of the genome with low expression activity including heterochromatin and quiescent regions. But, at stage four of the infection regions of the genome harboring HIV-related methylation changes are enhancers. We further showed that the effect of each of the virus subtypes on host methylome is to a large extent similar. And both virus subtypes appear to induce hypomethylation of loci associated with key pathways involved in viral infection such as ‘type I interferon signaling pathway,’ 'innate immune response' or 'negative regulation of viral genome replication.' Nevertheless, our results also indicate that each of the virus subtypes at least to some extent affects host methylome in virus-subtype-specific manner. Lastly, we showed that infection progression-related methylation changes that we identified are reversed with antiretroviral therapy.

**Conclusions:**

We have shown that progression of HIV infection is associated with hypomethylation of enhancers regardless of virus subtype. This suggests that methylation changes at the enhancers may be key for infection progression. However, we also identified methylation changes indicating that, each of the virus subtypes affects host methylome in specific manner, but these findings need to be confirmed in studies that include larger number of participants.

**Supplementary Information:**

The online version contains supplementary material available at 10.1186/s13148-025-01910-3.

## Background

After over 40 years since first HIV-related acquired immunodeficiency syndrome case (AIDS) was reported, progress in antiretroviral therapy (ART) has turned what was once a deadly infection into chronic and manageable disease. However, several conditions, such as non-AIDS-related diseases associated with chronic inflammation attributed to HIV, accelerated aging, and lack of full immune recovery despite effective antiretroviral therapy, impact length and quality of life of people living with human immunodeficiency virus (PLWH) [1–3]. 

Genome-wide association studies (GWAS) of PLWH have identified genetic variants affecting infection risk as well as dynamics of HIV disease progression including dominant impact of variants within *HLA* or *CCR* loci (as reviewed in: [4]). Nevertheless, the strength of the identified genetic interactions remains to be fully elucidated, especially in large population-based studies. Thus, as so far no general evidence for strong population wide association of host genetics with the outcomes of HIV infection has been identified, the significance of epigenetic changes induced by the virus in the host cells for the infection outcomes has become of interest for the researchers.

First studies of HIV-related methylation changes found that, for example, promoter of *FOXP3* gene involved in immune system responses was significantly less methylated in colonic mucosa and blood of PLWH in comparison to healthy controls [5]. In another study, *IL2* promoter was found completely methylated in naive CD4^+^T cells and demethylated in memory CD4^+^T cells during infection [6]. These studies were followed by epigenome wide methylation studies (EWAS) assaying entire host methylome for the virus-related methylation changes. One of the first of EWAS studies reported methylation changes at 20 CpG loci in blood cells of PLWH, which were not present in non-infected controls [7]. Majority of those changes were near or within genes known to be involved in immune activation. This study was followed by a number of studies which uniformly identified methylation changes associated with molecular pathways known to be essential for the viral infection (e.g., interferon signaling pathway). However, the identified methylation changes were frequently discrepant between studies [8–11]. Recently, acceleration of the epigenetic age of PLWH was associated with physiologic frailty and all-cause mortality [12] indicating that infection-related methylation changes not only accelerate epigenetic aging but also seems to be associated with clinical outcomes. 

Overall, changes in host methylome appear to play significant role in HIV infection pathology. However, so far methylation changes in hots epigenome during infection progression have not been studied in virus subtypes specific manner. For this reason, we analyzed methylomes of white blood cells of healthy individuals and methylomes of PLWH stratified by disease stage as well as virus subtype, with inclusion of two common European HIV-1 subtypes: subtype B (most common in the Western and Central Europe) and subtype A6 dominating in the Eastern Europe including Ukraine and Russia where HIV epidemics remains uncontrolled.

## Results

### HIV infection and progression are associated with DNA methylation changes in specific genomic regions

**Fig. 1:**
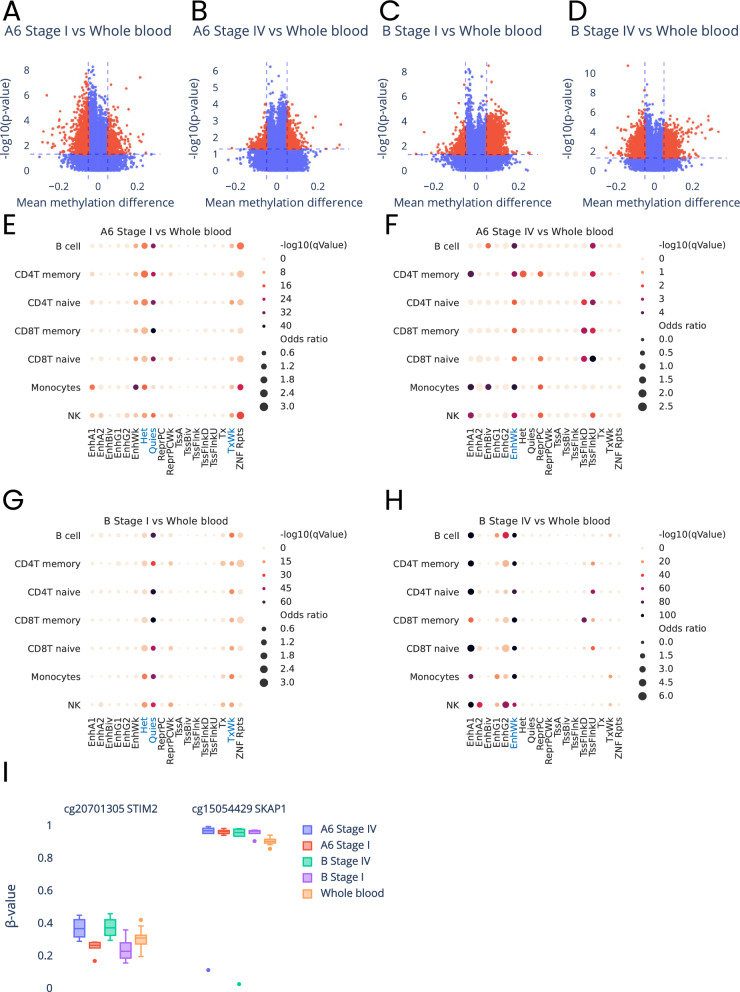
Characteristics of methylation changes at differentially methylated CpG sites identified between blood cells of PLWH and healthy controls for each of the virus subtype and infection stage. **A**–**D** Volcano plots illustrating methylation levels (beta-value) differences versus statistical significance $(-{\text{log}}_{10}\text{p-value})$ of observed methylation changes, at CpG sites identified in comparison of blood cells from healthy donors and: **A** blood cells infected with subtype A6 at stage I of the infection, **B** blood cells infected with subtype A6 at stage IV. **C** blood cells infected with subtype B at stage I and **D** blood cells infected with subtype B at stage IV. Statistically significant differences are marked in red. **E**–**H** Graphical illustration of association of genomic regulatory regions (according to 18-state model) in seven blood cell types, with differentially methylated CpG sites identified between: **E** blood cells from healthy donors cells and blood cells infected with virus subtype A6 at stage one, **F** blood cells from healthy donors cells and blood cells infected with subtype A6 at stage four, **G** blood cells from healthy donors cells and blood cells infected with subtype B at stage one, **H** blood cells from healthy donors cells and blood cells infected with subtype B at stage four. The regions displaying statistically significant associations are marked in blue. **I** Box plots illustrating methylation levels at two CpG sites common between all comparisons of methylomes of blood cells from healthy donors cells with the methylomes of blood cells from PLWH for each virus subtype and infection stage

We first compared methylomes of blood cells of healthy individuals with methylomes of blood cells from patients with stage one and four of the infection separately for two virus subtypes (A6 and B). This analysis identified a number of DMPs (Differentially Methylated Positions) that displayed statistically significant (*p*-value ≤ 0.05) methylation changes of more than five percent points (Fig. 1A–D and detailed description of the identified DMPs are shown in Supplementary Tables 1, 2, 3, 4). Then, to assess the potential physiological function of identified methylation changes, we used Locus Overlap Analysis (LOLA) and mapped identified methylation aberrations to genomic regions that included: chromosomes, CpG island elements (CGI, S-, N-shores, S-, N-shelfs and Opensea) as well as gene elements (TSS200, TSS1500, 1st exon, ExonBnd, 3’UTR, 5’UTR, gene body and intergenic regions). We found no specific enrichment of HIV-related methylation changes in any of analyzed regions. However, analysis of association of the identified methylation aberrations with histone marks in seven types of blood cells from Roadmap Epigenomics 18-state model [13] (Fig. 1E–H) showed relatively uniform results. Specifically, the methylation changes identified in stage one, regardless of virus subtype, were statistically significantly (FDR-corrected *p*-value ≤ 0.05) associated in all analyzed cell types with regions of weak transcription (TxWk), inactive chromatin states such as constitutive heterochromatin (Het) and quiescent state (Quies) (Fig. 1E and G and details of this analysis in Supplementary Tables 5 and 7). However, with the progression of the infection to stage four (AIDS), the genomic region harboring infection-related methylation aberrations changed to weak enhancers (EnhWk) indicating that methylation changes those elements are essential in virus progression and both analyses for each of the virus subtypes showed similar results (Fig. 1F and H and details of this analysis in Supplementary Tables 6 and 8). We then used GREAT platform to perform gene set enrichment analyses (GSEA), which allows to approximate the cellular processes potentially affected by the identified methylation changes. This analysis resulted in different number of the GO biological processes linked to DMPs identified in each of the four methylome comparisons performed here (the lists of the identified GO biological processes are shown in Supplementary Tables 9, 10, 11, 12). Interestingly, however, seven of those processes were common between majority of the analyses. The common processes included physiological pathways specifically related to the physiology of viral infection, including: 'innate immune response,' 'negative regulation of viral genome replication,' 'regulation of viral genome replication,' 'response to interferon-alpha,' 'response to interferon-beta,' 'response to type I interferon' and 'type I interferon signaling pathway.' These results show that the main cellular processes disturbed by virus-related methylation changes in infected cells are those essential for the infection progression and general host response to the infection.

The methylation changes that we identified between blood from healthy donors and each of the infection stages for two types of the virus appear to affect similar cellular processes; however, only three CpG sites were common between these four analyses (Fig. 1I). Interestingly, however, two of those CpG sites cg15054429 and cg20701305 were annotated to the bodies of genes with a very specific function in immune system: *SKAP1* (Src Kinase-Associated Phosphoprotein 1) and *STIM2* (Stromal Interaction Molecule 2), respectively. *SKAP1* encodes T cell adhesion and degranulation-promoting adaptor protein that plays a critical role in inside-out signaling by coupling T-cell antigen receptor stimulation to the activation of integrin (as described in: GeneCards ID: GC17M048133). *STIM2* (Stromal Interaction Molecule 2) has been shown to be involved in regulation of calcium concentrations in the cytosol and endoplasmic reticulum as well as activation of plasma membrane Orai Ca(2+) entry channels (as described in: GeneCards Id: GC04P026859). Most interestingly in mice *STIM2* gene has been shown to be essential component of CD4T and CD8T-dependent immune response against viral infection [14].The analysis of direction of methylation level changes at these CpG sites (Fig. 1I) showed that methylation at CpG site associated with *SKAP1* gene was higher than in blood from healthy donors at all stages of infection, and the methylation levels at CpG site annotated to *STIM2* increased most significantly with the infection progression. These results suggest that changes of methylation at CpG site associated with *SKAP1* take part in infection regardless of the infection stage. Contrary to *SKAP1* gene the methylation changes associated with *STIM2* gene appear to take part in infection progression because methylation levels at CpG sites associated with this gene differed between infection stages.

**Fig. 2:**
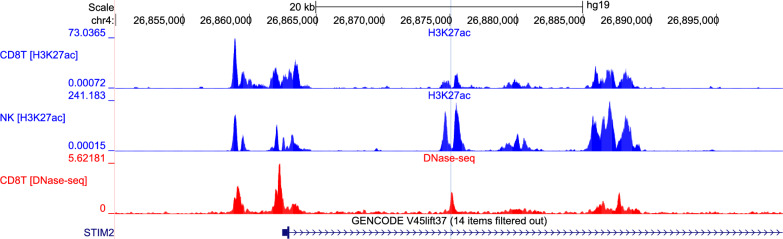
The overview of the locus (chr4:26,849,949–26,899,948) located in *STIM2* gene body harboring cg20701305 CpG site. The position of CpG site is indicated with vertical blue line, across three Genome Browser tracks illustrating from the top: occupancy of H3K27ac histone in CD8T and in NK cells as well as the map of the DNase hypersensitive regions on CD8T cells

Most interestingly, cg20701305 was not only located in *STIM2* but also in the enhancer that is predicted to interact with *STIM2* promoter (according to GeneHancer database [15]). Moreover, region harboring this CpG site varies with occupancy of H3K27ac (associated with higher activation of transcription and therefore defined as an active enhancer mark) between blood cell types as well as in DNase hypersensitivity site (Fig. 2). These results directly indicate potential regulatory function of methylation changes at this CpG site.

### Progression of HIV subtype A6 and B infection is associated with hypomethylation of enhancers

**Fig. 3:**
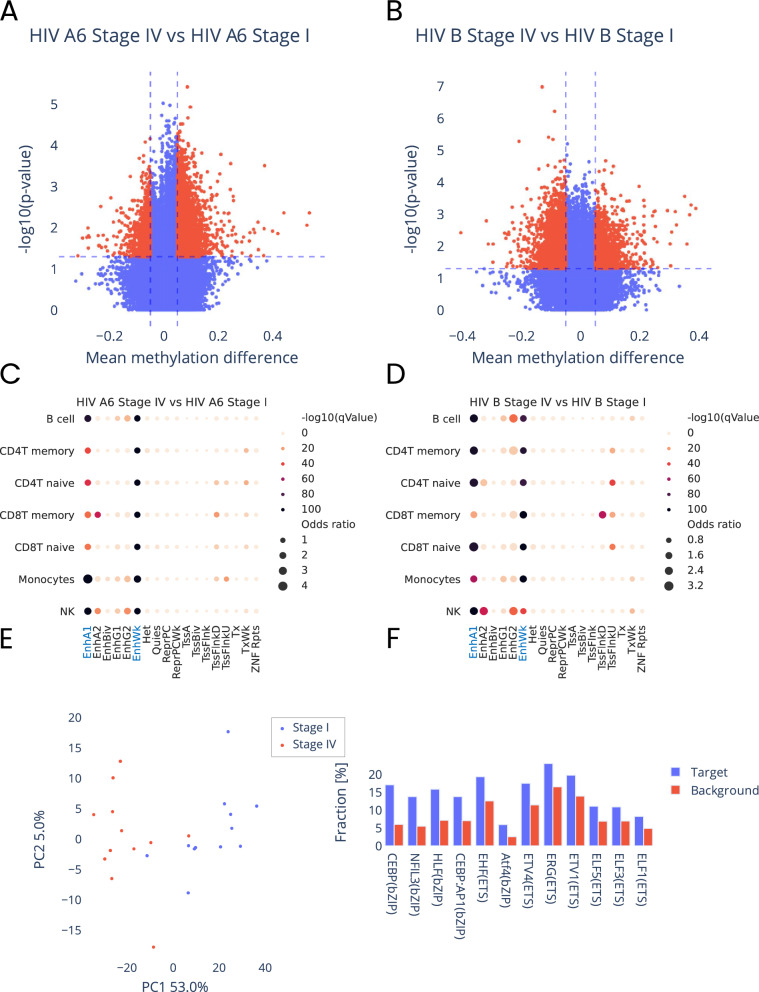
Characteristics of methylation changes at differentially methylated CpG sites identified between stage I and IV of the infection for each of the virus subtypes. **A**, **B** Volcano plots illustrating methylation levels (beta-value) differences versus statistical significance $(-{\text{log}}_{10}\text{p-value})$ of observed methylation changes, at CpG sites identified in comparison of: **A** blood cells infected with subtype A6 and **B** subtype B. Statistically significant differences are marked in red. **C**, **D** Graphical illustration of association of genomic regulatory regions (according to 18-state model) in seven blood cell types, with differentially methylated CpG sites identified between stage I and IV of the infection for virus subtype: **C** A6 and **D** B. The regions displaying statistically significant associations are marked in blue. **E** PCA plot based on methylation levels at CpG sites associated with the infection progression and common for both virus subtypes, showing distinct clusters of samples for each of the infection stages. **F** Graphical illustration of the results of transcription factor motifs enrichment analysis (HOMER) for regions harboring CpG sites associated with the infection progression and common for both virus subtypes. Bar plot illustrates percentage of CpG sites on the entire microarray targeted by specific TF (red bar) versus percentage of CpG sites targeted by specific TF identified in this analysis (blue bar)

Next, we compared methylomes of blood cells from I and IV stage of the infection separately for each of the virus subtypes. This analysis identified 8987 and 8030 methylation changes for virus subtype A6 and B, respectively (Fig. 3A, B and detailed description of the identified DMPs are shown in Supplementary Tables 13, 14). Here, we also used Roadmap Epigenomics 18-state model to assess the association of the identified methylation aberrations with histone marks in seven types of blood cells. This analysis showed that identified methylation changes were enriched (FDR-corrected *p*-value ≤ 0.05) in enhancer regions, specifically active enhancers (EnhA1) and weak enhancers (EnhWk) (Fig. 3C, D and the details of this analysis are shown Supplementary Tables 15, 16). This results again suggest that methylation changes at the enhancers are essential for the infection progression.

Interestingly, 578 of CpG sites identified in this analysis were common for both virus subtypes (detailed description of these DMPs is shown Supplementary Table 17). The PCA analysis based on this subset of CpG sites confirmed that the methylation levels at those CpG sites clearly differ between infection stages (Fig. 3E). And with the progression of infection majority of these CpG sites become hypomethylated (examples of DMPs from this analysis are shown in Supplementary Fig. 1). We also used HOMER transcription factor (TF) binding domain analysis to assess what type of transcription factors potentially bind to the loci harboring these methylation changes (Fig. 3F and details of the analysis in Supplementary Table 18). The TF binding sites enriched (FDR-corrected *p*-value ≤ 0.05) at these loci were mainly: Basic leucine zipper (bZIP) transcription factors including: CEBP(bZIP) shown to be required for HIV-1 replication [16], NFIL3(bZIP) that has been shown to be involved in T-cell dysfunction during viral infection or Atf4(bZIP) expression induction of which has been shown to increase with the replication of HIV-1 [17]. Moreover, in general, several cancer-causing viruses and HIV have been shown to modulate activity of TFs from bZIP and ETS families [18, 19]. We additionally performed GREAT analysis based on this subset of CpG sites. Not surprisingly, GO Biological ontology terms linked to the genomic regions harboring those methylation changes were associated with the molecular pathways essential for the infection progression (ontology terms from this analysis are listed in Supplementary Table 19). Finally, we analyzed the direction of the methylation change at 59 CpG sites linked in GREAT analysis to ‘type I interferon signaling pathway’ ontology term. This analysis showed that these CpG sites are enriched (FDR-corrected *p*-value ≤ 0.05) in promoter sequences of genes involved in this pathway (Supplementary Fig. 2A, B) and predominantly displayed lower methylation levels at stage four of the infection than the methylation levels at stage one (Supplementary Fig. 2C). These results along with the results of analysis of the direction of methylation changes at the differentially methylated CpG sites associated with the infection progression and common between virus subtypes indicate that hypomethylation of the genomic regulatory sequences is essential for the virus progression.

### Different subtypes of HIV virus appear to also induce subtype-specific methylation changes

**Fig. 4:**
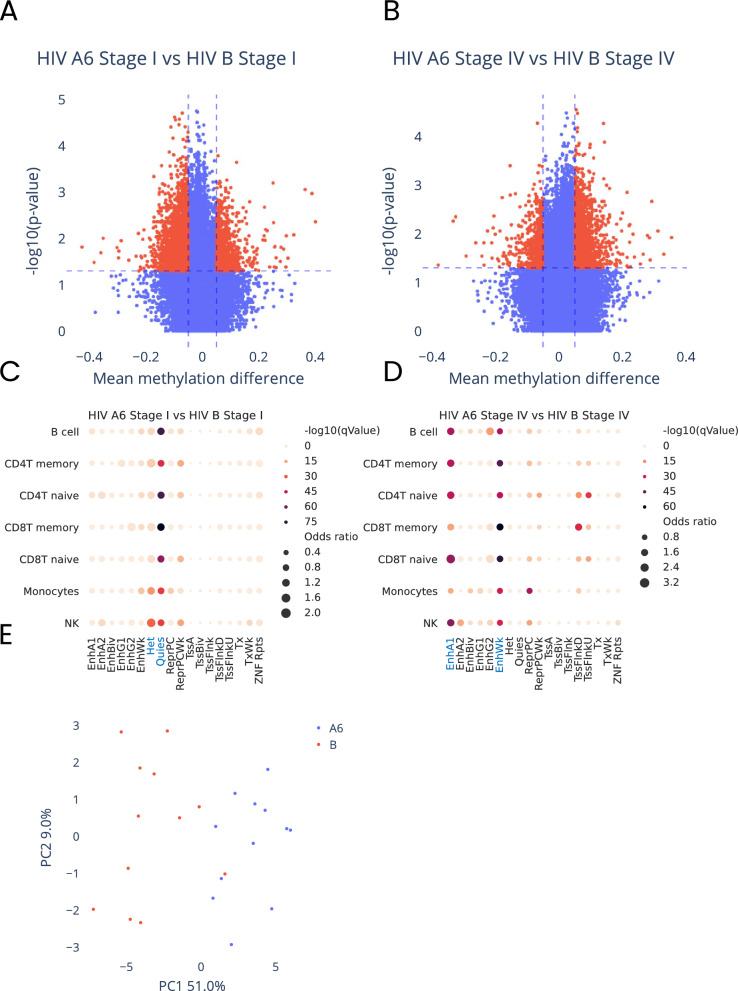
Characteristics of methylation changes at differentially methylated CpG sites identified between virus subtypes A6 and B at stage I and IV of the infection. **A**, **B** volcano plots illustrating methylation level (beta-value) difference versus statistical significance (− log10(*p*-value) of observed methylation changes, at CpG sites identified in comparison of: **A** blood cells infected with subtype A6 and B at stage I of the infection, **B** blood cells infected with subtype A6 and B at stage IV of the infection. Statistically significant differences are marked in red. **C**, **D** Graphical illustration of association of genomic regulatory regions (according to 18-state model) in seven blood cell types, with differentially methylated CpG sites identified between virus subtype A6 and B at **A** stage I and **B** stage IV of the infection **E** PCA plot based on methylation levels at differentially methylated CpG sites identified between virus subtypes A6 and B at stage I and IV of the infection, showing distinct clusters of samples for each of the virus subtypes

The data variability we observed in the analysis of methylation changes between virus subtypes and blood from healthy donors could suggest virus specific effect on host methylome. Thus, to identify potential virus specific methylation changes, we compared methylomes of blood cells from PLWH infected with each of the virus subtypes at stage I and IV of the infection. Surprisingly, this analysis identified 5965 and 3699 DMPs for virus subtype A6 and B, respectively, at each of the stages of the infection (Fig. 4A, B and detailed description of the identified DMPs are shown in Supplementary Tables 20, 21). These results indicate that despite generally similar effect of virus subtypes on host methylome, each of the virus subtypes appears to at least to some extent affects the host methylome in virus-subtype-specific fashion. Virus-subtype-specific DMPs identified at stage I of the infection were enriched (FDR-corrected *p*-value ≤ 0.05) in hetero (Het) and quiescent (Quies) regions of the chromatin and these identified at stage IV of the infection again in enhancers (EnhA1, EnhWk) (Fig. 4C, D, details of this analysis in Supplementary Tables 22, 23).

Among methylation changes that differed methylomes of patients infected with subtype A6 and B of the virus at each of the infection stages, 30 DMPs were present uniformly at stage I and IV of the infection (detailed description of these DMPs Supplementary Table 24). Majority of these methylation changes were annotated to the genes with established function in immune system including: *TAP2* which mediates translocation of peptide antigens from cytosol to endoplasmic reticulum (ER) for loading onto MHC class I (MHC I) molecules, *TRIM2* which is a protein involved in innate immunity against different DNA and RNA viruses or *PLCG1* associated previously with immune dysregulation, autoimmunity, and autoinflammation (source GeneCards). Interestingly, the PCA (Fig. 4E) as well as direct comparison of the methylation levels at these CpG sites stratified over virus subtype (Supplementary Fig. 3) clearly showed that the levels of methylation at those CpG sites not only differ between virus subtypes but also the direction of methylation changes at these CpG sites was different depending on the virus subtype. These results show that contrary to the methylation levels observed at the CpG sites common between two virus subtypes and almost uniformly becoming hypomethylated during infection progression (Supplementary Fig. 2), virus-subtype-specific methylation changes may induce different physiological effect. But this needs to be further confirmed in better powered studies.

### Identified methylation changes in the context of previous analyses of methylomes of PLWH and efficiency of ART

One of the first studies that reported methylation changes between blood cells of people living with HIV and healthy controls was study [7]. In that study authors found 20 CpG sites at which methylation levels were statistically significantly different between blood from PLWH and healthy controls. In our analysis only methylation levels at cg07839457 annotated to *NLRC5* gene displayed statistically significant methylation changes between blood methylomes of PLWH and healthy controls (Supplementary Fig. 4).

To further validate our results in independent cohort of PLWH, we have identified three other studies where methylome of blood cells of PLWH were assessed [20, 21] but no raw data files (.idat) were available from those studies to allow validation of our results. We were, however, able to access raw data from the study [22] that included methylation profiling data from blood of the healthy individuals as well as PLWH samples before and after administration of ART therapy. After pre-processing data from that study with our analysis pipeline, data from: 169 pre-ART, 166 post-ART and 44 healthy controls have passed our quality control assessment. We then compared the methylation levels at the CpG sites at which methylation levels in our study were associated with infection progression regardless of virus subtype. This analysis showed that 19 of those CpG sites displayed methylation level changes between pre- and post-ART patients (Supplementary Table 25). The direct comparison of methylation levels at those CpG sites between pre- and post-ART patients showed that the therapy reverses methylation changes at those CpG sites to the levels observed in blood from healthy donors cells (Supplementary Fig. 5A) which is the opposite direction of methylation change from the one observed during infection progression. Moreover, the STRING [23] analysis that allows to identify known protein–protein interaction (PPI), linked 13 out of 14 proteins encoded by genes harboring identified methylation changes into one network (Supplementary Fig. 5B) comprising interferon-related elements, and even more importantly the MAD-5 protein (encoded by *IFIH1* gene) being innate immune receptor for intron-containing RNA HIV-1 provirus [24].

## Discussion

Previous studies of influence of human immune deficiency virus on host methylome uniformly identified virus-related methylation changes in blood cells of PLWH. However, the influence of the HIV on host methylome during infection progression, especially considering specific virus subtypes, has not been studied so far. In our study, we analyzed methylation changes in host methylome during infection progression with two common European HIV-1 subtypes: subtype B (most common in the Western and Central Europe) and subtype A6, dominating in the Eastern Europe including Ukraine and Russia where HIV epidemics remains uncontrolled.

Our results show that majority of the methylation changes we identified in blood of the individuals infected with virus subtype A6 and B appear to affect similar cellular processes involved in immune response to viral infection and genes with established role in the immune system such as *SKAPT1*. Most interestingly, however, we found that, regardless of virus subtype in the beginning of the infection (stage I), virus-related methylation changes more frequently affect parts of the genome with low expression activity such as heterochromatin and quiescent regions. However, after infection progression to stage IV, genomic regulatory elements that harbor infection-related methylation changes are occupied by chromatin marks specific for enhancers. This suggests that methylation changes at enhancers are key for the progression of HIV infection for both subtypes of the virus. Importantly, we were able to confirm key role of methylation changes at enhancers during infection progression with detailed analysis of methylation changes that we linked to *STIM2* gene which is one of the key genes involved in infection progression and general host response to virus. Methylation change that we identified in this gene was located in the enhancer. To our best knowledge, in previous studies, the enhancer regions have been identified as only one of many other genomic regions displaying methylation changes upon HIV infection [25, 26]. Our results indicate that methylation changes at the enhancers may play a key role in the infection progression. Especially that methylation has long been shown to regulate the function of these important regulatory elements [27].

Interestingly, our analyses were also able to identify a number of the virus-subtype-specific methylation aberrations. This suggests that despite the major effect of virus on the cells is similar but there are also methylation changes that only specific virus subtype induces. These virus-subtype-specific methylation alterations also appear to affect enhancers and map to the genes involved in immune system physiology. 

Due to the obvious limitation of our study including low number of samples in each of the comparison groups, we focused our analysis on the methylation changes that are common between different comparisons we performed. This approach appears to at least to some extent overcome the imitations of our study, especially that using data from independent study, we have shown that methylation changes we identified appear to be reversed by ART therapy. This shows that despite of limitations, our study allowed for the identification of biologically and potentially clinically sound methylation changes that should be further elaborated.

## Conclusions

We have shown that in the beginning of the infection subtype B and subtype A6 of human immunodeficiency viruses affect parts of host blood cells methylome with low expression activity, including heterochromatin and quiescent regions, whereas with the infection progression the genomic regulatory elements that harbor infection-related DNA methylation changes are enhancers. Moreover, our results indicate that in general both subtypes of the virus in similar manner affect the host methylome. However, we also shown that each of the virus subtypes at least to some extent appears to induce virus-subtype-specific methylation changes in host methylome.

## Materials and methods

### Study population

The study included 24 antiretroviral treatment naïve people with HIV (mean age = 38, SD = 7.15 years, 62% male), enrolled in the Department of Infectious, Tropical Diseases and Acquired Immunodeficiencies, Pomeranian Medical University, Szczecin, Poland, representing four equally sized (*n* = 6) groups stratified based on virus subtype (A6 and B) and WHO HIV disease stage (I/HIV and IV/AIDS). All samples were collected according to the protocol approved by the institutional review boards with written consent obtained from each patient (KB-0012/118/04/19). We also used microarray profiles from 24 adults (mean age = 24, SD = 8.16, 42% males) with no symptoms of any disease, available at GSE222927 as a control cohort in our study.

### DNA extraction and initial preparation

Genomic DNA was extracted from peripheral blood samples using QIAamp DNA Mini Kit (QIAGEN GmbH, Hilden, Germany). The quantity and quality of obtained DNA were assessed using Qubit™ dsDNA BR Assay and Qubit® 2.0 Fluorometer (Invitrogen, MA, USA). Bisulfite conversion of DNA (500 ng) was carried out using EZ DNA Methylation-Gold Kit (Zymo Research, CA, USA), according to the manufacturer protocol.

### HIV subtyping

For HIV-1 RNA isolation, reverse transcriptase, and protease sequencing, the ViroSeq HIV-1 Genotyping System v 2.9 (Abbot Molecular, IL, USA) was used. Amplicons obtained by the nested PCR method were used for sequencing by standard techniques with BigDye technology on an ABI 3500 platform (Applied Biosystems, Foster City, CA). For subtype assignment, sequences were initially assessed using COMETv2 [28] and confirmed by phylogenetic analyses. For phylogenetic inference, sequences were aligned with Clustal Omega software [29]. A GTR + I + G model with four gamma categories was selected as optimal for the analyzed dataset. There were no recombinant subtypes within the sample.

### Genome-wide methylation analysis

Before methylation profiling all samples, groups were randomized and uniformly distributed over the slides to be able to address potential technical artifacts. Methylation profiling was performed using Infinium MethylationEPIC BeadChip (Illumina Inv., CA, USA). QC of raw idat files was performed using MethylAid package [30]. The data were processed using ChAMP pipeline and corrected for tissue composition (as described in [31]). Statistical analyses were performed using t test, Welch's *t* test or Mann–Whitney U test, depending on data distribution. The data distribution for each CpG was assessed using Shapiro–Wilk and Levene tests. Differentially methylated positions (DMPs) were defined as CpGs with significant methylation difference (*p*-value ≤ 0.05) and absolute average methylation level difference between compared groups ≥ 0.05.

### Functional analyses

Genomic context enrichment analysis was performed using Genomic Locus Overlap Enrichment Analysis (LOLA) package [32] and custom databases generated using: Illumina manifest annotations (Relation_to_Island, RefGene_Group and CHR) as well as 18 types of genomic segments predicted based on six histone marks (H3K4me3, H3K4me1, H3K36me3, H3K27me3, H3K9me3, H3K27ac) for white blood cells (samples id: E039, E040, E048, E047, E029, E032, E046), downloaded from 18-state model [13]. To test the statistical significance of enrichment, we used default one-sided Fisher exact test. Functional analysis of genes harboring these CpGs was performed using GREAT (Genomic Regions Enrichment of Annotations Tool) version 4.0.4 tool, which allows to predict functions of cis-regulatory regions. Analysis of transcription factor (TF) motif enrichment was performed using the findMotifsGenome.pl script from HOMER [33], with the following HOMER specific parameters: hg19 reference genome, mask repeats/lower case sequence, CpG normalization, and hypergeometric test. Due to asymmetrical distribution of CpG sites targeted by EPIC microarrays all enrichment analyses were adjusted for a background comprising all CpGs available in this experiment. ChIP-seq as well as DNase-seq tracks for white blood cells were extracted from ENOCDE database (ENCFF214ZJG, ENCFF363KIC, ENCFF026UTG).

### Quantification and statistical analysis

Python version 3.10 and R version 4.1.2 were used for statistical analyses. The statistical significance level assumed in this study was equal to 0.05. All analyses were conducted using hg19 reference genome.

## Supplementary Information

Additional file1 (XLSX 6602 KB)

## Data Availability

The raw data generated in this project are available under accession number: GSE272115.

## Abbreviations

PLWH: People living with human immunodeficiency virus
ART: Antiretroviral therapy
GSEA: Gene set enrichment analysis
DMP: Differentially methylated position
DMR: Differentially methylated region
TssA: Active transcription start site
TssFlnk: Flanking transcription start site
TssFlnkU: Flanking transcription start site upstream
TssFlnkD: Flanking transcription start site downstream
Tx: Strong transcription
TxWk: Weak transcription
EnhG1: Genic enhancer 1
EnhG2: Genic enhancer 2
EnhA1: Active enhancer 1
EnhA2: Active enhancer 2
EnhWk: Weak enhancer
ZNF/Rpts: ZNF genes & repeats
Het: Heterochromatin
TssBiv: Bivalent/poised transcription start site
EnhBiv: Bivalent enhancer
ReprPC: Repressed polycomb
ReprPCWk: Weak repressed polycomb
Quies: Quiescent/low
